# Successful Tuberculosis Treatment Outcomes among HIV/TB Coinfected Patients Down-Referred from a District Hospital to Primary Health Clinics in Rural South Africa

**DOI:** 10.1371/journal.pone.0127024

**Published:** 2015-05-19

**Authors:** Karen B. Jacobson, Anthony P. Moll, Gerald H. Friedland, Sheela V. Shenoi

**Affiliations:** 1 Icahn School of Medicine at Mount Sinai, New York, New York, United States of America; 2 Church of Scotland Hospital, Tugela Ferry, KwaZulu-Natal, South Africa; 3 Department of Medicine, Section of Infectious Diseases, AIDS Program, Yale University School of Medicine, New Haven, Connecticut, United States of America; Harvard Medical School, UNITED STATES

## Abstract

**Background:**

HIV and tuberculosis (TB) coinfection remains a major public health threat in sub-Saharan Africa. Integration and decentralization of HIV and TB treatment services are being implemented, but data on outcomes of this strategy are lacking in rural, resource-limited settings. We evaluated TB treatment outcomes in TB/HIV coinfected patients in an integrated and decentralized system in rural KwaZulu-Natal, South Africa.

**Methods:**

We retrospectively studied a cohort of HIV/TB coinfected patients initiating treatment for drug-susceptible TB at a district hospital HIV clinic from January 2012-June 2013. Patients were eligible for down-referral to primary health clinics(PHCs) for TB treatment completion if they met specific clinical criteria. Records were reviewed for patients’ demographic, baseline clinical and laboratory information, past HIV and TB history, and TB treatment outcomes.

**Results:**

Of 657(88.7%) patients, 322(49.0%) were female, 558(84.9%) were new TB cases, and 572(87.1%) had pulmonary TB. After TB treatment initiation, 280(42.6%) were down-referred from the district level HIV clinic to PHCs for treatment completion; 377(57.4%) remained at the district hospital. Retained patients possessed characteristics indicative of more severe disease. In total, 540(82.2%) patients experienced treatment success, 69(10.5%) died, and 46(7.0%) defaulted. Down-referred patients experienced higher treatment success, and lower mortality, but were more likely to default, primarily at the time of transfer to PHC.

**Conclusion:**

Decentralization of TB treatment to the primary care level is feasible in rural South Africa. Treatment outcomes are favorable when patients are carefully chosen for down-referral. Higher mortality in retained patients reflects increased baseline disease severity while higher default among down-referred patients reflects failed linkage of care. Better linkage mechanisms are needed including improved identification of potential defaulters, increased patient education, active communication between hospitals and PHCs, and tracing of patients lost to follow up. Decentralized and integrated care is successful for carefully selected TB/HIV coinfected patients and should be expanded.

## Background

Tuberculosis (TB) remains a major public health threat worldwide, with 8.6 million new cases diagnosed in 2012[1]. The HIV epidemic has significantly contributed to the rising TB prevalence particularly in sub-Saharan Africa. South Africa ranks fifth in the world in TB incidence, and first in number of TB/HIV coinfection cases with 65% of TB patients coinfected with HIV[1]. In South Africa’s hardest-hit province, KwaZulu-Natal (KZN), TB incidence approaches 1100 per 100,000 population.

Historically, TB and HIV programs have operated separately as vertical treatment models. However, integration of TB and HIV services has been identified as a way to improve diagnosis and treatment for both HIV and TB[2]. Screening for TB in HIV patients, and, conversely, screening for HIV in TB patients yields increased case finding and earlier diagnosis and linkage to treatment[3]. Concurrent co-trimoxazole prophylaxis and antiretroviral therapy (ART) during TB treatment improves survival and treatment outcomes for HIV patients[4,5], and having co-located treatment delivery systems improves dual treatment provision for both providers and patients[6].

Tuberculosis diagnostic and treatment services in vertical programs have long been successfully decentralized to the community level in many resource-poor settings[7–10], and separate decentralization of ART provision has been rolled out within the past decade in many areas of South Africa and neighboring countries[11–14]. Initial studies of decentralized HIV services only have shown success, with high ART coverage rates[12], better patient retention in treatment[13,15], and low mortality[11,12,16]. However, when integrated TB/HIV services have decentralized to the primary care level, results have been mixed[3,9,17,18]. While some sites have shown that integrated services at the primary care level increase diagnostic yield for TB, increased coverage of HIV testing in TB patients, and decreased time to ART initiation for patients on TB treatment, it is unclear whether TB treatment outcomes are favorably affected[17,19].

We sought to evaluate drug-susceptible TB treatment outcomes in HIV-positive patients who initiated TB treatment in an integrated TB/HIV treatment setting at a rural district hospital and either remained at the secondary district hospital care level or were down-referred to the primary health clinics for continued TB/HIV integrated services.

## Methods

### Study Setting

Tugela Ferry is located in the Msinga subdistrict of rural KwaZulu-Natal, South Africa, home to approximately 180,000 traditional Zulu people. Poverty is endemic with an unemployment rate of 85 percent, and only approximately 39 percent of the population has access to electricity and 31 percent to clean water[20]. The Church of Scotland Hospital (COSH) is a 350-bed provincial government district hospital located in Tugela Ferry, and serves the population of Msinga along with 16 nurse-managed satellite primary health clinics (PHCs) which refer patients to COSH for secondary level care and to which COSH down-refers patients for primary care services, including ART and TB treatment[21]. Patients may access TB diagnostic services including sputum microscopy and culture at COSH as well as all PHCs. Routine GeneXpert MTB/RIF testing was implemented at COSH in 2013. The outpatient HIV clinic at COSH offers integrated HIV and TB treatment services for HIV-positive patients.

Patients initiating ART or TB treatment at the COSH HIV clinic may be down-referred, when stable, for treatment continuation at a PHC closer to their place of residence. This practice is in compliance with the National Tuberculosis Management Guidelines (2009) stating that clients should be down-referred when medically stable, without dyspnea, hemoptysis, severe emaciation or fever[22]. Practitioners at the COSH HIV clinic down-refer patients who are clinically improving; ambulating well; with stable cardiovascular, respiratory and nutritional status; without serious adverse effects from medication; and who have demonstrated good adherence, and observed insight and understanding of treatment expectations including perceived community and family support (Table 1). Down-referral may occur at any point during TB treatment, according to provider judgment and patient preference. Patients recommended for down-referral are instructed to present to the PHC clinic within two weeks of their final COSH visit. Social work services are not available. Patients are given a referral letter to bring to the PHC; no other formal communication occurs between COSH and PHCs informing them of step-down referral and no standard mechanisms exist for tracking those who do not turn up at PHCs.

**Table 1:** Current specific criteria for down-referral implemented at the district hospital HIV Clinic.

Patients eligible for down-referral to PHC if following conditions are met
● Patient ambulating well
● Showing clinical improvement
● No serious medication adverse effects
● Stable cardiovascular, respiratory, and nutritional status
● Observed insight and understanding of treatment expectations including perceived community and family support
● Patient prefers to access treatment in the community
● History of good medication adherence

### Ethics Statement

The South African Medical Association Research Ethics Committee and Yale University School of Medicine Human Investigation Committee approved the study and granted a waiver of informed consent to conduct the record review. Patient records were de-identified prior to analysis.

### Data Collection

Records of patients initiating TB treatment at the district hospital’s HIV clinic between January 1, 2012 and June 30, 2013 were reviewed retrospectively. Data was abstracted from the HIV clinic’s standardized KwaZulu-Natal Department of Health tuberculosis treatment register, including basic demographics, HIV history, TB history, TB diagnosis and treatment outcomes, and, when incomplete, was supplemented by review of patient charts and electronic pharmacy records. Treatment outcomes for patients who were subsequently down-referred to PHCs were obtained from review of the TB treatment registers at the PHCs, using South African identification numbers to identify patient records. Patients were excluded from analysis if they had drug-resistant TB, transferred out of the district, or were still undergoing treatment.

### Data Analysis

Data was anonymized prior to analysis. Descriptive statistics were employed to characterize the patient population, including differences between those retained at district hospital level and those down referred. TB treatment outcomes were defined according to the 2009 South African National Tuberculosis Guidelines. “Cure” was recorded for patients with negative sputum smear in the last month of treatment and on at least one previous occasion >30 days prior. “Treatment completed” was recorded for patients who completed the duration of treatment but do not meet criteria for “cured” or “treatment failure”. “Treatment success” was defined as either cure or treatment completion. “Treatment failure” occurred if the patient remained smear-positive at 5 months of treatment. A patient who died for any reason during TB treatment was recorded as “Died”. “Defaulters” were patients whose treatment was interrupted for more than two consecutive months[22]. Chi-square and t-tests were used to examine differences between patients who remained at the district hospital HIV clinic for the duration of their TB treatment and patients who were down-referred to PHCs. Data were analyzed using SPSS 22.0.

## Results

**Fig 1:**
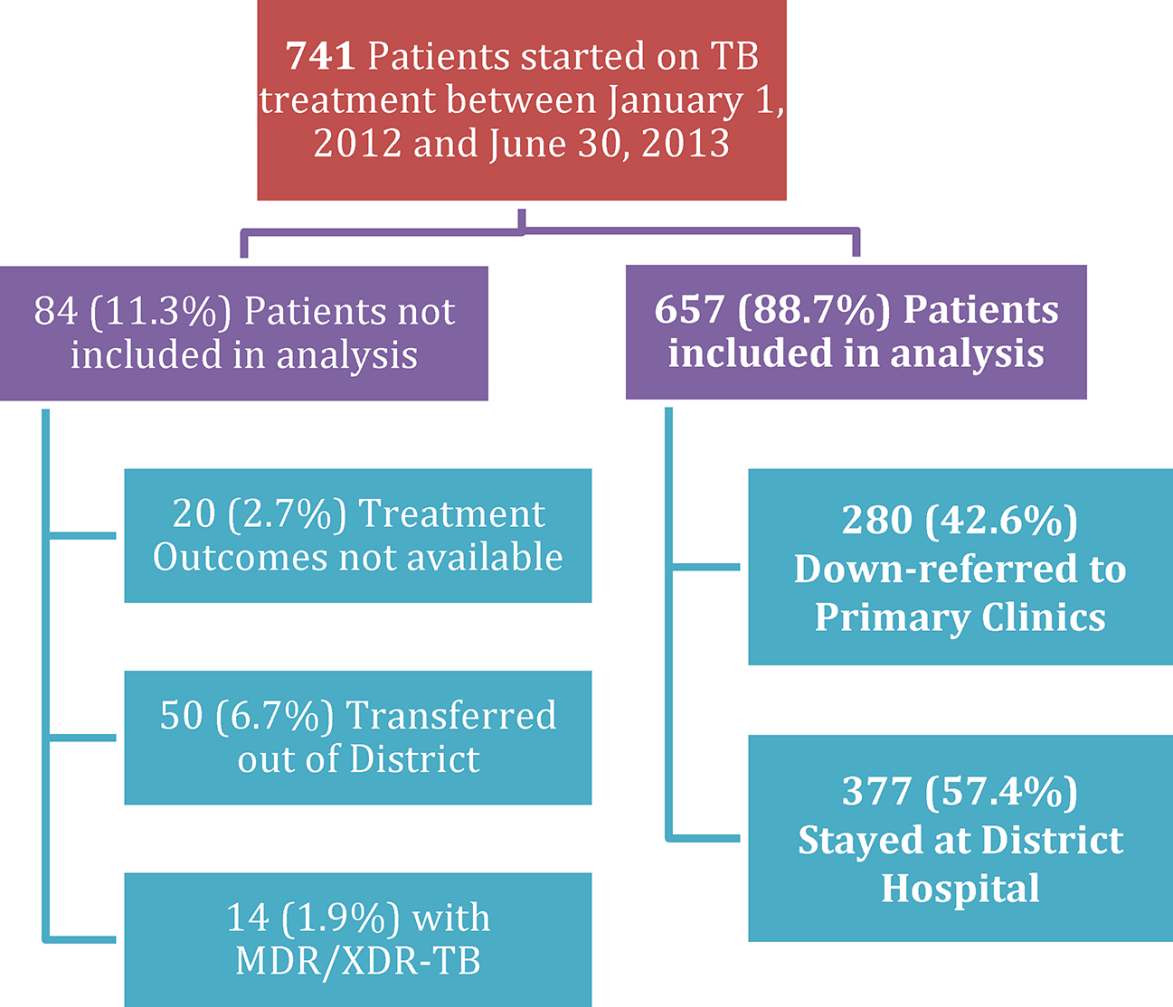
HIV-infected Patients initiating TB Treatment at District Hospital. Seven hundred forty-one patients began tuberculosis (TB) treatment at the district hospital between January 1, 2012 and June 30, 2013. Patients who were still on treatment at the time of data collection, who transferred care to another district, or who were diagnosed with multiple drug resistant (MDR) or extensively drug resistant (XDR) TB were not included in data analysis. Of 657 patients with available treatment outcomes for drug susceptible TB, 377 remained at the district hospital for TB treatment and 280 were down-referred to complete their TB treatment at primary health clinics in their communities.

A total of 741 patients initiated TB treatment at the COSH HIV clinic between January 1, 2012 and June 30, 2013. Of these, 657 (88.7%) had available treatment outcomes; 50 (6.7%) transferred to another district, 20 (2.7%) had not completed treatment at the time of data analysis, and 14 (1.9%) had MDR or XDR-TB and were not included in analysis (Fig 1). Among the 657 patients included in the analysis, nearly half (49.0%) were female, with median age 34 (Interquartile Range (IQR) 26–42); 84 (12.8%) were under age 15, 558 (84.9%) were new TB cases, 572 (87.1%) had pulmonary TB, and 73 (11.1%) had extrapulmonary disease (Table 2). The majority of diagnoses were made clinically according to South African TB Guidelines[22]; only 79 (12.0%) cases were diagnosed based on microbiological data with a positive AFB smear, culture, or GeneXpert MTB/RIF.

**Table 2:** Characteristics of all patients, those remaining at district hospital, and down referred to primary care clinics.

Parameter	All n = 657 unless otherwise noted	District Hospital n = 377	PHC n = 280	p value
Age (Median)	34.0 (26–42)	33.0 (25–41)	35.5 (28–43)	0.07
Proportion female	322 (49.0%)	46.7%	52.1%	0.17
CD4 (Median) [Adults ≥ 15 yrs only]	123 (50–227)	106.0 (39–205)	136.0 (62–256)	0.03
New TB Case	558 (84.9%)	315 (83.6%)	243 (86.8%)	0.25
Pulmonary TB cases (n = 655)	572 (87.3%)	325 (86.7%)	247 (88.2%)	0.56
AFB Smear Positive (n = 617)	42 (6.8%)	35 (9.9%)	7 (2.7%)	<0.01
Culture positive (n = 327)	64 (19.6%)	43 (22.8%)	21 (15.2%)	0.09
GeneXpert MTB/RIF positive (n = 13)	3 (23.1%)	3 (37.5%)	0 (0.0%)	0.27
TB diagnosis microbiologically confirmed with positive AFB, Culture or GXP^1^	79 (12.0%)	57 (15.1%)	22 (7.9%)	0.01
On ART prior to TB treatment	176 (26.8%)	106 (28.1%)	70 (25.0%)	0.42

^1^GeneXpert MTB/RIF testing became available beginning in 2013.

In total, 540 (82.2%) patients completed treatment or were cured, 69 (10.5%) died during treatment, and 46 (7.0%) defaulted. One patient had treatment stopped due to medication hepatotoxicity, and one patient failed treatment. Overall, mortality was significantly higher in men than women (14.9% versus 6.5%, p = 0.001; Odds Ratio (OR) 2.6 (95% CI 1.5–4.5)) and in patients with CD4<200 than with CD4 > = 200 (13.9% versus 4.7%, p<0.001; OR 3.2 (95% CI 1.7–6.3)). Among patients who died, median time to death was 38 days after starting TB treatment (IQR 13.5–84.5) and 19 of 69 (27.5%) deaths occurred within 2 weeks of treatment initiation. Patients were more likely to default if they were not on ART at the time of TB treatment initiation (9.2% versus 4.5%, p = 0.049) or had previously defaulted from TB treatment (21.4% versus 7.3%, p = 0.007). TB disease characteristics, including AFB smear or culture positivity and extrapulmonary disease, were not associated with treatment success, death, or default.

Two hundred eighty (42.6%) patients were down-referred to PHCs to complete TB treatment. Compared to patients who were down-referred to PHCs, adult patients who remained at the district hospital’s HIV clinic to complete TB treatment had a significantly lower median CD4 count (106.0 versus 136.0, p = 0.03) and were more likely to have microbiologically confirmed tuberculosis with AFB, culture or GeneXpert MTB/RIF (15.1% versus 7.9%, p = 0.002). Children under 15 years were less likely to be down-referred (20.2% of children versus 46.9% of adults, p<0.001). There were no differences in gender between those who were down-referred and those remaining at the district hospital HIV clinic. Among patients remaining at COSH, 78.5% achieved treatment success, 16.7% died, and 4.2% defaulted, and among down-referred patients 87.1% achieved treatment success, 2.1% died, and 10.7% defaulted (Table 3). In the down-referred cohort, patients who were down-referred within one month of treatment initiation were less likely to default compared to those down-referred later (6.0% versus 15.4%, p = 0.012).

**Table 3:** Treatment outcomes of all patients, those remaining at district hospital, and down referred to primary care clinics.

Outcome	All Patients	District Hospital n = 377	PHC n = 280	p-value
Duration of TB treatment (median days and IQR)	196 (181–241)	204(174–245)	187(182–234)	<0.01
Treatment Completion	472 (71.8%)	245 (65.0%)	227 (81.1%)	<0.01
Cure	68 (10.4%)	51 (13.5%)	17 (6.1%)	<0.01
Cured or Completed	540 (82.2%)	296(78.5%)	244 (87.1%)	<0.01
Defaulted	46 (7.0%)	16 (4.2%)	30 (10.7%)	<0.01
Mortality	69 (10.5%)	63 (16.7%)	6 (2.1%)	<0.01

**Fig 2:**
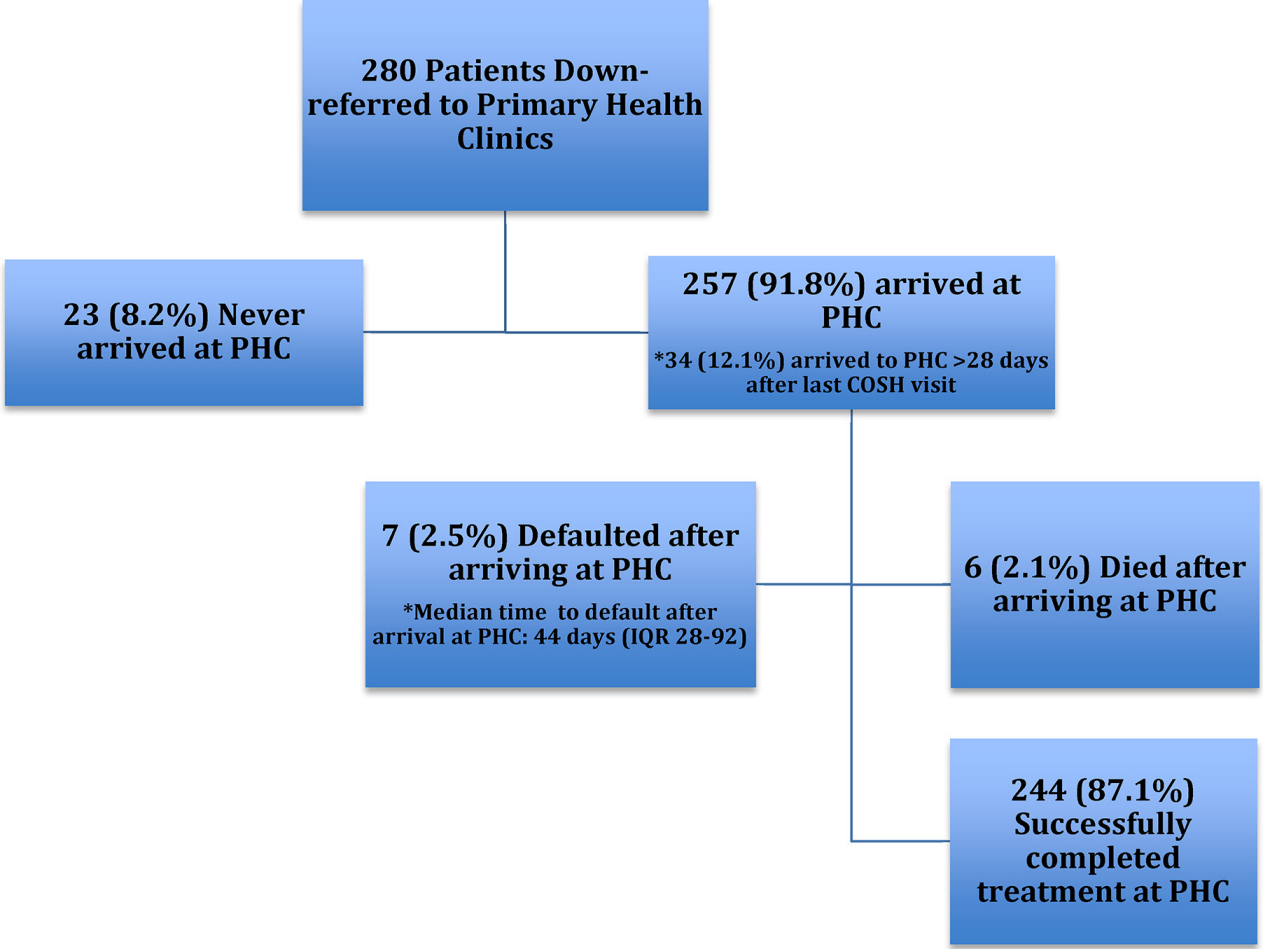
Timing of default in down-referred cohort. Of 280 patients who were down-referred from the district hospital clinic to primary health clinics (PHCs) for completion of tuberculosis (TB) treatment, 23 (8.2%) never arrived at a PHC. Of those that did successfully link to a PHC, 34 (12.1%) arrived late and thus missed doses of TB medication; 7 (2.5%) defaulted before completing TB treatment; 6 (2.1%) died while on TB treatment; and 244 (87.1%) successfully completed treatment at the PHC.

Of the 280 patients who were down-referred to PHCs, 100 (35.7%) were down-referred immediately after the initiation of TB treatment at the district hospital HIV clinic, and 48% of patients were down-referred within 30 days of beginning TB treatment. The median time from down-referral (last district hospital visit) to initial PHC visit was 7 days (IQR 1–17); of 216 patients with documented PHC arrival dates, 120 (55.6%) patients linked to PHC care within one week, and 183 (84.7%) linked within 28 days of their last centralized care site visit. Twenty-three (8.2%) of down-referred patients had no record of successful linkage, accounting for 76.7% of the 30 defaulters in the down-referred cohort (Fig 2).

## Discussion

In this retrospective study of a single rural district hospital experience in TB and HIV treatment integration and decentralization, we evaluated TB treatment outcomes among HIV patients in an integrated treatment setting who either remained at the district hospital level or were down-referred to decentralized primary health clinics. We have made a number of important observations relevant to the recommended strategies of integration and decentralization of TB and HIV particularly in rural, resource-limited settings.

Overall, this cohort of HIV patients beginning TB treatment at the district hospital level had an 82.2% treatment success rate (completion or cure), which is excellent but falls slightly short of the national goal of >85%[22]. One in ten patients died, with mortality being strongly associated with low CD4 count (<200 cells/mm3). The majority (>70%) of these TB patients were not on ART at the time of TB treatment initiation, indicating that TB remains an important reason for HIV-infected individuals to enter HIV care and treatment. This also emphasizes that even in the era of expanded ART eligibility for patients with CD4 < 350 (as were the guidelines at the time these patients were in treatment), many patients were still not accessing ART early enough to prevent opportunistic infections and improve survival. Previously, in nearby Hlabisa, KZN, Barnighausen et al found that, only 72% of eligible patients were receiving ART in 2010 based on current guidelines at that time[23]. Identifying patients earlier in the course of their disease through community based intensive case finding and voluntary counseling and testing programs, and ensuring that all individuals start in a timely manner is essential to helping reduce TB mortality in coinfected patients[5]. South Africa has now implemented a threshold of CD4<500; facilitating ART initiation at this earlier stage will likely positively impact TB incidence and thus mortality.

Only 12% of patients in this cohort had microbiologically confirmed TB, which is consistent with a review of TB diagnoses performed in another KZN district in 2005 that found that only 15% of patients were initiated on treatment based on a positive sputum sample[21]. More recently, in the EXTEND trial of GeneXpert MTB/RIF, the availability of rapid diagnostics did not significantly improve survival as clinicians empirically initiated TB therapy at very high rates among TB suspects pending GeneXpert MTB/RIF results[24]. This highlights the need for both improved point of care diagnostics for pulmonary and extrapulmonary TB and implementation research regarding the most effective use of new TB diagnostics in resource-poor settings.

There was a high rate of default among TB/HIV coinfected patients down-referred to PHCs. The majority of the defaulters in the down-referred cohort were lost to follow up at the time of linking to PHCs, never arriving at their designated PHC after their last district hospital appointment (Fig 2). This loss to follow up during referral is a problem that has been reported in settings where patients are referred for TB or HIV treatment initiation [25] or down-referred from hospital to PHC level for continued ART provision[26,27], but not for continuing TB care. It is possible some of the patients in our study continued their TB treatment at a different PHC than the one to which they were originally referred; this problem of incomplete tracking patients for treatment continuation has been observed in other resource-poor settings[27,28]. Patients in our study were not found to be receiving TB treatment at neighboring PHCs within the district, but some patients may have relocated outside the district to their place of work or family home and could potentially be accessing care outside the district. The high failed linkage rate is concerning because patients who do successfully link to PHC care have excellent treatment outcomes, representing a lost opportunity for successful continuation and completion of care. Patients failing to continue TB treatment potentially transmit TB to others in the community and propagate the epidemic. In our study, of the patients who did successfully link, 85% arrived at the PHC within 4 weeks of their last district hospital visit. Since patients are either given 4 weeks or 2 weeks of medication per visit, this suggests that at least 15% had a gap in treatment (Fig 2). Of note, patients were more likely to default if they were not on ART at the time of TB treatment initiation or had previously defaulted from TB treatment, suggesting the utility of more careful evaluation of such patients before down-referral.

Down-referred patients were less likely to default if down-referral occurred earlier during the course of treatment. We do not have direct evidence of why this might be the case, and see this as an important area for study. When eligible for down referral, patients should be carefully counseled on the need to continue treatment at the PHC and the required duration of treatment. This and other indicators of down-referral success require further prospective study and validation as predictors of outcomes.

Improved health systems are needed to assist patients in successfully linking to the primary care level without delay. Currently, no formal procedures exist for predicting who might default or for tracking patients who have been referred to PHCs and ensuring follow-up. The South African national TB management guidelines recommend that all patients referred to the clinic level be accompanied by a social worker or DOTS supporter, be delivered to or collected by the clinic, and when this is not feasible, that follow-up should be performed to ensure patient linkage[22]. Thus, formal communication between PHCs and district hospitals should take place regarding referred patients and mechanisms to trace patients who fail to link should be developed similar to tracing other defaulters. These measures should include utilizing tracing teams and local community health workers (CHWs) to locate patients[29,30] establishing a communication network through which PHCs can communicate with each other directly in addition to communicating with secondary health facilities, and using unique patient identifier numbers within services at each site (i.e. same for both ARV and TB treatment registers). In addition, recommendations from ART decentralization may be applicable to TB treatment in this setting; these include standardizing patient identification numbers between PHCs and district hospitals, and training clinic staff to educate patients on the down-referral process[28]. Patients should be carefully interviewed before down-referral to ascertain their travel intentions and establish their preferred point of follow-up, so, if necessary, transfer can be arranged in the case of relocation out of the district to their place of work or family home.

Coinfected patients down-referred to PHCs had a favorable TB treatment completion rate on par with district hospital clinic patients. Even when taking into account the high numbers of patients lost during linkage, more down-referred patients completed treatment, and among down-referred patients who actually arrived at the PHCs there were very low death rates and default rates. The low proportion of default is comparable to findings among HIV patients successfully down-referred from hospital to primary clinic level for ART continuation in other settings in South Africa and in Malawi[13,16]. Though HIV treatment outcomes were not evaluated in the current study, the high TB treatment success rate demonstrates that patients remained in care with opportunity for continued HIV treatment in an integrated primary care setting.

Adult patients remaining at the district level had lower CD4 counts, were more likely to have positive microbiological confirmation of TB with either AFB, culture, or GeneXpert MTB/RIF, and were more likely to die. These predictors likely reflect the tendency for providers to use clinical judgment to identify patients at highest risk of morbidity and mortality. This tendency to down-refer less sick patients is similar to the ART decentralization experience in other resource-poor settings[13,28]. The direct comparison of the down-referred group and the retained group must take into account the limitation that those eligible for down-referral are less ill and thus more likely to experience treatment success; however, the adequate TB treatment outcomes and low death rates among the down-referred cohort compared to the retained cohort suggest that specific clinical criteria for down referral can successfully identify patients who are well enough to benefit from down-referral. Thus, if the down-referred patient population is chosen carefully, they are likely to experience adequate treatment outcomes at the PHC level. However, these criteria have not been prospectively validated as a reliable tool to determine which TB/HIV patients are stable enough to down-refer or would be more likely to default is not presently available. Clinical severity scales have been developed to predict outcomes in drug susceptible TB patients and mortality in XDR-TB patients[31,32], though these have not been used for determining which patients would benefit from retention at the centralized facility versus down-referral. Future scales validated prospectively in drug-sensitive TB patients would provide more definitive data to guide practitioners in determining whether patients are stable enough for down-referral, and for predicting default during the down-referral process.

Integration of TB and HIV treatment services, and decentralization of these services to the primary care level, has been advocated as a means of improving outcomes for both TB and HIV in resource-limited settings. In this cohort of TB/HIV coinfected patients in a decentralized and integrated treatment program, overall TB treatment outcomes were excellent. Outcomes can be further enhanced by improving linkage to care in down-referred patients and further improved by increasing access to ART before active TB disease through earlier identification via community screening, and initiating ART in all TB/HIV coinfected patients[5].

This study has several limitations characteristic of retrospective studies. All data were dependent on paper registers kept in the district hospital and the primary health clinics, which were not uniformly complete. Though we attempted to locate information missing from the registers using other hospital record sources, this was not always possible. The study period included the rollout of GeneXpert MTB/RIF diagnostic technology, for which there was not yet an official data column in the registers, and therefore information about GeneXpert MTB/RIF may not be complete. Although severity of HIV disease is documentable through CD4 cell counts, no single available measure of severity of TB disease is available and thus TB severity was not well characterized. Regarding documentation of ART status, patients may have initiated ART in the past and subsequently defaulted, and this information was not uniformly documented. Additionally, though the vast majority of patients not already on ART were initiated on ART after TB treatment initiation, this was not uniformly documented in the TB registers and thus the impact of subsequent ART initiation on TB outcomes could not be estimated. Furthermore, it was not possible in this retrospective study to document the uniformity of practices regarding down-referral criteria, and therefore we can suggest but cannot confirm that these were implemented.

## Conclusion

HIV patients can be successfully down-referred to the primary care level for continuation of TB treatment, as long as they meet certain clinical criteria and successful linkage is ensured. When deciding who is eligible for down-referral, medical practitioners should carefully consider the patient’s clinical status, history of medication adherence, and previous treatment default or failure, and the patient’s preference and ability to follow up at community primary health clinics versus the district level. Patients deemed eligible for down-referral require counseling on the need for continuing treatment and careful attention to the vulnerable period immediately following referral. Future research goals should include development of reliable methods of predicting which patients can be successfully down-referred and a means of tracking patients to ensure successful linkage to primary care.

## Supporting Information

S1 File TB in HIV Clinic Dataset. This file contains the dataset of n = 657 patients initiating TB treatment between January 1, 2012 and June 30, 2013 at the Church of Scotland District Hospital clinic discussed in this manuscript.
(XLS)
